# What is it like to use a BCI? – insights from an interview study with brain-computer interface users

**DOI:** 10.1186/s12910-019-0442-2

**Published:** 2020-01-06

**Authors:** Johannes Kögel, Ralf J. Jox, Orsolya Friedrich

**Affiliations:** 1 Institute of Ethics, History and Theory of Medicine, LMU Munich, Lessingstr. 2, 80336 Munich, Germany; 2 Clinical Ethics Unit and Institute of Humanities in Medicine, Lausanne University Hospital and Faculty of Biology and Medicine, University of Lausanne, Avenue de Provence 82, CH-1007 Lausanne, Switzerland; 3 Institute of Philosophy, Faculty of Cultural and Social Sciences, FernUniversität in Hagen, Universitätsstr. 33, 58097 Hagen, Germany

**Keywords:** Brain-computer interfaces, Neuroethics, Empirical research, User experience, Agency, Self-image, Participation

## Abstract

**Background:**

The neurotechnology behind brain-computer interfaces (BCIs) raises various ethical questions. The ethical literature has pinpointed several issues concerning safety, autonomy, responsibility and accountability, psychosocial identity, consent, privacy and data security. This study aims to assess BCI users’ experiences, self-observations and attitudes in their own right and looks for social and ethical implications.

**Methods:**

We conducted nine semi-structured interviews with BCI users, who used the technology for medical reasons. The transcribed interviews were analyzed according to the Grounded Theory coding method.

**Results:**

BCI users perceive themselves as active operators of a technology that offers them social participation and impacts their self-definition. Each of these aspects bears its own opportunities and risks. BCIs can contribute to retaining or regaining human capabilities. At the same time, BCI use contains elements that challenge common experiences, for example when the technology is in conflict with the affective side of BCI users. The potential benefits of BCIs are regarded as outweighing the risks in that BCI use is considered to promote valuable qualities and capabilities. BCI users appreciate the opportunity to regain lost capabilities as well as to gain new ones.

**Conclusions:**

BCI users appreciate the technology for various reasons. The technology is highly appreciated in cases where it is beneficial in terms of agency, participation and self-definitions. Rather than questioning human nature, the technology can retain and restore characteristics and abilities which enrich our lives.

## Background

Brain-computer interfaces (BCIs) are increasingly becoming the focus of public and scientific attention. The public interest in BCIs has expanded even more since the well-known entrepreneur Elon Musk co-founded the company Neuralink, whose research focuses on BCIs. BCIs are neurotechnological devices that detect and measure brain activity and convert these brain signals into computer-generated output, which can consist of various tasks such as moving a cursor, operating computer programs, steering a wheelchair, controlling prostheses or activating muscles [1–5]. The output serves as real-time feedback that the users receive, may it be visual, auditory, tactile, vestibular or proprioceptive [2]. The brain signals can be measured invasively or non-invasively, most commonly an electroencephalography (EEG) is used by placing electrodes on the surface of the skull [2, 3]. Three types of BCIs can be distinguished according to the brain activity production that is being used [6].

Active BCIs require the user to perform a mental task, which is then used as a command for the BCI application. The mental strategy that is often used in these BCIs is motor imagery, i.e. the user imagines moving a body part. The recorded brain signal is then translated into a specific BCI output. Imagining closing your hand may lead to a robotic arm grasping a cup [7].

Reactive BCIs operate via the mental strategy of selective attention. They make use of brain activity that occurs as a result of an individual’s voluntarily focused attention on a specific external stimulus. A commonly used paradigm for reactive BCIs is the P300 setting, which can detect which stimuli the user is concentrating on. The stimuli may be characters or icons displayed on a screen. This setting is used, for instance, in the Brain Painting application [8].

Passive BCIs do not require a voluntarily performed task. The user’s brain activity is simply measured when it is confronted with certain tasks. Passive BCIs are used to examine cognitive states like mental workload or attention, or affective states [6].

BCI applications may fall in the medical arena or the consumer arena. Within the medical arena, BCIs are often intended to restore or increase communication and motor skills for persons with physical impairments, improve rehabilitation for persons who have had a stroke or spinal cord injury, and help to regulate or treat persons with psychiatric conditions or epilepsy [9–13]. The consumer arena consists of BCI applications such as gaming, entertainment and enhancement [14, 15].

These technologies potentially raise interesting questions that ethicists have highlighted and discussed. Burwell et al. [16] synthesize ethical issues addressed throughout the BCI literature. They comprise safety, risk-benefit deliberations, humanity and personhood, stigma, normality, autonomy, responsibility, informed consent and research ethics, privacy, security and justice. Scoping socio-empirical studies on BCIs, Kögel et al. [17] show that most studies aim to assess the acceptance of BCIs among users and to improve the performance and quality of the technology. Empirical studies with BCI users with physical impairments that employed qualitative research methods, such as qualitative interviews or focus groups, assess some points that reach beyond the medico-technological evaluation of BCIs. Users report benefits in terms of increased independence and autonomy [18], experiencing happiness and enjoyment [10, 19–22], as well as valuing the opportunity for creativity and self-expression [20–22] and self-experience [23]. The possibility of social participation and communication was positively rated [24], whereas the additional workload for their caregivers [18] and their dependence on others [21] was considered worrisome.

Thus far, little empirical research has been done that explicitly addresses the philosophical or psychosocial aspects of BCI use. By taking the value and importance of the perspective of users with physical impairments into consideration, we aim to explore the subjective perspective of medical BCI users. As BCI actions are achieved by bypassing the peripheral nervous system, acting via a BCI may feel and be different from operating another technology.

The research question of this study is how BCI users with physical impairments perceive using a BCI with regard to the following notions: (1) Self-experience: How does it feel to operate a BCI? What effects does BCI use have on bodily sensations and self-image/−understanding? (2) Interactions: What am I able to do with a BCI? How do other people react towards me as a BCI user? (3) Evaluation: Does a BCI benefit me currently or will it benefit me at some point in the future? Does a BCI enable and empower or does it rather alienate and increase dependence?

Following the methods section, we will give a brief overview of the study participants. Next, we will outline the major themes or categories that emerged from the interviewees’ accounts (being an agent, participation, self-definition). Eventually, the categories will be discussed in relation to each other and the literature.

## Methods

An interview study was performed in order to explore how users perceive BCI use. Due to the explorative design of our research that focuses on the subjective dimensions of BCI users, we chose a qualitative socio-empirical approach to our study. Nine semi-structured interviews were conducted with BCI users. The prerequisite criterion to be included in our sample was having used a BCI for medical purposes, i.e. having participated in BCI studies for medical research that recruited persons with physical impairments. Another inclusion criterion was that the BCI had to be one that was operated, i.e. active or reactive BCIs. We were interested in what it is like to operate a BCI and what it is like to cause actions with it. Therefore, passive BCIs were ruled out. In order to recruit participants for our study, we approached target groups and patient organizations and contacted BCI research teams that run experimental BCI studies. The approach via target groups turned out to be unsuccessful in that the number of people who participated in BCI studies proved to be too insignificant. A gatekeeper effect limited our access to contact details to only a handful of BCI users. Further participants were recruited through contacts from BCI users whom we interviewed.

Three interviews were conducted at users’ homes. One participant preferred to answer the interview questions in written form, due to speech impediments. In the case of two further participants with oral speech impediments, responses in written form were combined with visits at their home. Three interviews with participants living in other countries were conducted via online video call. All interviewees declared informed consent for their participation in the interview study before the interviews commenced.

Three interviews (Robert, Neil and Nicole) were held in English. The others were conducted in German. Excerpts from the German transcriptions that are displayed in this article as interview citations were translated into English by the authors.

The topic guide (see Additional file 1) for the interviews covered several themes: personal background (living conditions, independence/dependence on caregivers, role of technology), first contact and experiences with BCIs, action-theoretical aspects of BCI usage and general opinion on BCIs (benefits, risks, advantages, disadvantages, expectations, future scenarios).

The interviews were transcribed verbatim and the informal discussions with the participants outside of the interview were written down as field notes. The transcripts were analyzed using the coding approach outlined by Grounded Theory literature [25, 26]. In a first step, the transcripts were coded openly, i.e. data were broken down into conceptual components. In a second step, concepts that emerged from the first step were put in relation to each other giving rise to three categories (being an agent, participation, self-definition).

The coding procedure of Grounded Theory suits the explorative endeavor of our research. It allows for openness in terms of research interests, attentiveness and sensitivity of the researcher, while giving him tools to form groups of codes and to put emerging categories into relation, leading to theorizations which are still “grounded” in the data.

## Results

### Information on the study participants

The participants of the interview study were between 24 and 77 years old and were diagnosed with different muscular conditions (see Table 1). In terms of nationality, the sample comprised six participants from Germany, two from the US and one from France. The sample also varied in terms of gender, BCI type used and BCI experience. All of the participants have participated in experimental BCI studies before. One participant had a home-based BCI.

**Table 1:** Participants of the interview study (names have been pseudonymized)

Participants (name, age)	Diagnosis	Technologies used	BCI technology (*applications*)	Number of BCI (training) sessions
Stefan, 24	generalized dystonia	wheelchair, eye tracker, computer	NIRS-BCI (Near-Infrared Spectroscopy), non-invasive	1
Walter, 32	muscle atrophy	wheelchair, email/typing and voice recognition software, computer, respiration apparatus	P300-BCI (*email-Software, Brain Painting*), non-invasive	3
Wolfgang, 31	muscle atrophy	wheelchair, email/typing and voice recognition software, computer, respiration apparatus	P300-BCI (*email-Software, Brain Painting*), non-invasive	3
Karl, 46	Duchenne muscle dystrophy	wheelchair, email/typing software, computer, respiration apparatus	P300-BCI (*email-Software, Brain Painting*), non-invasive	Ca. 20
Mrs. Edlinger, 77	amyotrophic lateral sclerosis	wheelchair, email/typing software, computer, BCI	P300-BCI (*Brain Painting*), non-invasive	> 100 (ongoing)
Rudi, 27	tetraplegia	wheelchair, computer	MI-BCI (motor imagery) (*BrainRunners*), non-invasive	> 50
Robert, 51	paraplegia	wheelchair, computer	MI-BCI (+exoskeleton training), non-invasive	> 50 (ongoing)
Neil, 30	tetraplegia	wheelchair, email/typing and voice recognition software, computer	MI-BCI (+ robotic arm), invasive (implanted electrodes)	> 50 (ongoing)
Nicole, 58	spinocerebellar ataxia	wheelchair, email/typing and voice recognition software, computer, respiration apparatus	MI-BCI (+ robotic arm), invasive (implanted electrodes)	> 100

Stefan used a NIRS-BCI, however, the test session was not successful and thus he was not enrolled in the experimental BCI study. All of the other participants managed to operate a BCI successfully. Neil and Nicole had electrodes implanted on their primary motor cortex. The other participants used an EEG cap for BCI use.

Out of all the users who were able to successfully operate a BCI, only a few regarded BCIs as a viable alternative to the technologies that they were already using. Most of them are still in control of some body movements and exploiting these movements allows them to use more efficient technologies. Nevertheless, as we will see, some of these users were able to get some benefits out of BCI training, such as the opportunity for social participation. As a consequence, some users did not expect too much from BCIs, but still participated in the research studies either out of curiosity or because they regarded the research as important and wanted to play their part.

Hence, the hopes and expectations of users were quite mixed at the beginning of the BCI research studies. Accordingly, the users’ responses to BCI use varied. Some users were quite euphoric:

“[Y]ou couldn’t wipe the smile off my face, I was really thrilled.” (Nicole).

“It was really cool. It was like Fuckin A! I had such a smile on my face and I just thought ‘That’s incredible.’ That is so futuristic, you know. You thought that’s like a pie in the sky and you became kind of a pioneer in this field. That was really cool. No doubt, really really cool.” (Rudi).

Some users showed excitement:

“Yes, it was pretty cool. […] In the beginning it sounds futuristic, like science fiction, but it is no science fiction, but by now it is science – without fiction. And that’s a cool thing, I think. […] ‘What was it like?’ – Interesting and cool.” (Walter).

Other users had a neutral feeling towards BCI use:

“Well, during training I didn’t have any negative or positive feelings in particular. It was, I would say, neutral. In the beginning, perhaps, it was still exciting and interesting to see that it all works out, but after two to three sessions it was normal.” (Karl).

This heterogeneity among the study participants will also be shown in the following sections. While all users who managed to operate a BCI agreed on feeling like the agent while using it and most participants appreciated the opportunities of social participation, the aspect of self-definition reveals a wide range of different dimensions that play a part in the interviewees’ accounts. BCI use engages the users in some form of self-definition processes, which display a great variety in terms of reference points and experiences.

### BCI users’ self-perceptions

Three main categories emerged during the analysis of the interview transcripts: being an agent, participation and self-definition. All three categories are intertwined with each other in manifold ways. Prior to discussing the relations between the categories, the categories themselves and the various relations within the categories will be examined.

#### Being an agent

“I can make a machine do it.” (Nicole).

Using a BCI means being active.^1^ Users need to perform certain mental tasks, in most cases by either concentrating on particular stimuli or imagining various body movements [2, 6]. These can be more or less strenuous, both mentally and physically. That the users are active goes without saying. Whether the users also feel like they are the cause of the BCI-generated actions is a different question. Being active alone does not suffice in creating the feeling of being the agent of BCI-mediated actions.

Two aspects appeared that accompany the users’ feeling of generating the BCI output. One is the perception that the BCI activity that has been performed was successful, which means that the BCI generated the output that the BCI user had in mind. The other refers to a common, everyday life understanding of technology, meaning that technology is seen as a tool that we use to do something with and that this tool could not have performed the task without our doing.

Starting with the first aspect, it does not simply suffice that the BCI produces an objectively measurable output in order for it to be successful. Also, according to the subjective point of view of the user, the output needs to match the intention that the user had. One user expressed it in this way:

“Due to a high success rate, I had the feeling that I did execute the BCI activity myself, for example choosing an option in the painting software, and not the computer. Or, in other words, I did have the feeling that the computer or the program was doing what I wanted it to do.” (Karl).

This *conversion*,^2^ i.e. the feeling that the user’s intention is converted into the appropriate action, seems to be necessary for the users in order for them to feel like they are the agent of the BCI output. Apparently, a high enough success rate seems to be sufficient in evoking this feeling, as also indicated by another user:

“I was active. I could – well, it worked in about 95 per cent of the cases, let’s say, my command. In five per cent there was another command, but still, it was me who was active.” (Walter).

When asked whether the BCI implemented her intentions accordingly, one user answered:

“Definitely. Otherwise my doing would be in vain and pointless.” (Mrs. Edlinger).

The BCI output serves as the immediate feedback to the user, who is able to judge the success of the BCI actions “depending on whether the screen displays what I commanded to display” (Walter). The wanted output can be portrayed visually (on the screen), but also in the form of motions of an exoskeleton or a robotic arm.

In addition, BCI users have a particular understanding of technology and its usage, which mirrors the understanding we commonly hold and practice toward technology in our everyday life. As users of technology, we determine what to do with it and the technology is simply a tool for us to achieve a certain goal.

“Let me put it like this: it is like my computer. The computer SHOULD always be the extended arm. Hence, I am still the executive power. I am the brain in that sense (...). If I wouldn’t be in control of myself, the computer wouldn’t have anything to utilize. That’s how it is.” (Rudi).

“[I]t is us who control the machine. I don’t think it ever will be the other way around.” (Walter).

Given this *actor-centered understanding of technology*,^3^ it may seem obvious that we define ourselves as the agents of BCI use and may also contribute to assigning oneself agency over BCI actions.

Both aspects of successful BCI activity, the successful conversion and the actor-centered understanding of technology – which are exemplified in the quote cited above (“I can make a machine do it.” (Nicole)) – accompany the feeling of being the agent of the generated output, i.e. a *sense of agency*, defined as, “the sense that I am the one who is causing or generating an action” [27].

This perception creates a sense of responsibility for BCI-generated actions, which becomes apparent in particular in the face of unsuccessful BCI actions.

“When there was a wrong command, I got cross with myself, because I thought somehow ‘Oh, apparently I didn’t concentrate properly.’” (Walter).

“I always was annoyed that I may have focused too little. I felt responsible, yes, because the computer doesn’t do this on purpose or want to make me angry, but because it just can’t help it. So - it is on me.” (Wolfgang).

Both users blame themselves and their supposedly deficient concentration. Wolfgang cannot help but take the blame as the computer just seems to be obeying his commands. This again illustrates the actor-centered point of view on technology.

Allocating responsibility for unwanted BCI output also depends on the cause of unsuccessful actions. Accordingly, users make certain differentiations:

“It depends whether I voluntarily executed the activity or whether it happened, for example, due to an error in the system or by accident. For the former I naturally feel responsible, for the latter not quite.” (Karl).

While it is rather easy for the user to assume responsibility for successful BCI actions (“Yes, I, like, I know that I am actually doing them (…) Yes, I feel responsible for them” (Neil)), it gets tricky in the case of failed BCI actions. Sometimes users can identify possible causes for failed BCI actions.

One user, for example, talked about a training session where there was noise from a construction site in front of the building.

“I still remember the day when there was a construction site outside. […] a construction site with construction vehicles that were making noise and that day my measurements did not work very well. There just were more errors or there were more occasions when the computer registered something else than what I intended. That may be because of the noise of the site that distracted me.” (Wolfgang).

It is noteworthy that Wolfgang refers to “errors” which occurred. He didn’t say “I made mistakes.” Hence, he is neglecting responsibility for failed BCI actions in that context. On another occasion it is more difficult for him to pinpoint the causes for failed actions.

“Well, I guess I didn’t concentrate hard enough. But it also may be that the measuring was not optimal. […] Also, the more time the computer has, the better the probability that it detects the right things. Maybe time was simply short.” (Wolfgang).

At the beginning, Wolfgang blames himself for not concentrating hard enough. On second thought, there may have been technical problems such as the measuring or the time setting.

As concentration is pivotal to BCI use, distractions can be detrimental to successfully executed BCI actions. Sources of distraction can be external, like noise from a construction site, or they can be internal.

“I still remember my first training. I started to get to know somebody and then she said before the training ‘It’s not working out with the two of us.’ Well, then my thoughts were completely somewhere else and then trying to calm down – hence, logically that training session was shit. You have these influences. Or when my pet died – you naturally have these influences, every human does.” (Rudi).

Also, emotional states like excitement or fear were reported as sources for low success rates. In these cases, with either internal or external causes of distraction, low success rates were expected and thus do not challenge the link between agency and accountability.

However, the causes of failure were not always transparent to the users and often left room for uncertainties within the users’ accounts. For many users, unintended BCI output came as a surprise.

“Yes. IF there was a wrong command, it took me by surprise, yes.” (Walter).

“[…] it was quite a surprise, for instance, you don’t answer properly that means [I] was thinking on the right and it was the left and when it was like that I shouldn’t have-- I should really not care about, because if I care it’s even worse. That means that the next round, it goes wrong also. Next try sorry, it goes wrong because I have too much focus on why, why, why.” (Robert).

Here, Robert points to another difficulty of BCI use. A peculiar challenge is to not get worked up about incorrect BCI outputs, even when you know that you sent a different command, because getting emotional will have a negative impact on the commands that follow.

“I was NOT allowed to get upset, because that also would have been a signal and that would have influenced the training. You are NOT allowed to have emotions, none whatsoever. You completely have to be dead inside.” (Rudi).

This *imperative of no emotions* is a challenge for users in that emotions are a pivotal part of being human, but need to be suppressed or avoided during BCI use. Users report that they had to learn to fade out personal issues during BCI training and to really focus on the tasks at hand.

Other users ascribe failures to the technology.

“I always say brains are dumb, but really it’s sometimes computers are dumb at figuring out what your brains are thinking, so some days it’s, especially if I haven’t slept well the night before and I’m already tired, sometimes the stuff just doesn’t work super good. […] sometimes the, the programming isn’t that great. But yes, and then sometimes robots are super durable, sometimes they just wear down or overheat and so there are still many points of failure that are possible in these systems.” (Neil).

Even though Neil acknowledges his own tiredness, he can still identify possible flaws in the technology’s soft- and hardware. Another user reports:

“More often than not, they [the technical team] had to figure out what was wrong with the training, or what was wrong with the hardware or software because my brain was doing what it was supposed to do.” (Nicole).

In general, we can see that the ascription of accountability for unsuccessful BCI use oscillates between personal and technological issues leading to some uncertainty when it comes to attributing agency or accountability.

The level of uncertainty about the agency of (some) BCI activity may have two possible causes. It may stem from an uncertainty about the actual control one has over BCI actions. Assuming responsibility for successful actions while rejecting it in the case of failed attempts may refer to the phenomenon of “illusion of control.”^4^ This is also reflected in the vocabulary that users employ. “I did execute …” or “It was me who …” was used when referring to their BCI actions. When describing failed BCI actions, they used the third person and phrases like, “When there was a wrong command […].” This points to the second possible cause, which denotes an uncertainty of speech. BCIs are a relatively new neurotechnology. Hence, there is no established way to talk about these technologies in everyday life. This grey zone of language use may explain the users’ uncertainty. As we have seen, the matter of responsibility becomes contested as soon as we are dealing with unsuccessful and, especially, unintended BCI actions. There is no consent on this matter. While some BCI experts hold the view that users are indeed responsible for BCI-generated outcome [28], others claim that BCI use leaves room for uncertainty regarding one’s sense of agency or “judgement of agency” [29]. This room for uncertainty becomes apparent in the users’ account of the interview study.

Both the difficulty of agency perception and the difficulty of how to talk about BCIs may be grounded on an outdated understanding of advanced neurotechnology. Therefore, we may need to adopt (in accordance to our language of everyday life) a terminology that is used to describe technologies by means of “distributed agency” and “inter-agency” [30]. In this case, we need to accept that in advanced technologies human and non-human agents are intertwined in such a way that individual tasks of various agents become indiscernible. Hence, attributing responsibility for the overall output to a single agent appears problematic.

#### Participation

“EVERY human wants recognition.” (Rudi).

BCIs offer users various opportunities for social participation. One way is to include potential or actual BCI users in research and technology development processes [31–33]. Other studies have acknowledged the importance of social participation for users [21, 24]. BCIs are reported to increase independence [18] and to provide opportunities for creativity and self-expression [20–22].

Our interviewees appreciated having had the opportunity to contribute to research and developing medical technology. Hereby, BCI training was regarded as a *meaningful occupation *that also included *being part of a team*, receiving *social recognition* for what they were doing and *taking part in public life*.

“[T]hat’s good for me at least to be seen as a helpful person, although, I do understand that I’m only at the end of the chain and, but to feel I’m part of the team.” (Robert).

For Robert, it is important to be regarded as someone who contributes to scientific research. This is supported by the feeling of being a member of the research team. This team spirit is also important for other users.

“I miss the training with [the robotic arm]. What I miss even more is having the job. I went to the office three days a week. I had co-workers, people I worked with and became close to. I miss the camaraderie of working with the group. I miss having a place to go and something to do every day.” (Nicole).

For Nicole, this loss of the “job” (after the BCI research study came to an end) weighs more than the actual tasks that were performed using the BCI. The BCI training provided some kind of normal life of having a regular job, working in a team and having the social life that goes along with it.

Rudi also expresses these two aspects: team spirit and the meaning of the training as an occupation:

“Partly, I miss the training, definitely. I got out more often – that sounds funny – I got out of my place, which wasn’t possible without assistance. I had a lot of contact with people. I got to know many people also at the Cybathlon^5^ which was very very cool. I did get recognition for what I did […] Hence, I miss it, definitely. I would like – it would be nice if I could get the opportunity again to participate at the Cybathlon again in 2020 and to compete for a team. Everyone said the Cybathlon was made possible due to me, but I said ‘No, we are a team.’ A pilot is only as strong as his team and I had a strong team that supported me, were responsive to me, and were considerate of me. It was ALL of us together – that time really was extreme. We saw each other very often. […] We were like a small family.” (Rudi).

Hence, with the end of the study Rudi had to give up opportunities to socialize and lost his team or even “family”. The importance of team spirit and the mutual respect and recognition within the team is also stressed by Nicole.

“Just how honored I am to have been able to contribute to this and how it wasn’t just my contribution. […] People said, ‘Oh, look at that Nicole, she’s doing so great. She’s doing such wonderful things’ when it’s the result of the whole team. In this case, it took thousands of scientists to design the robotic arm, to figure out how the brain works, to make implants that would read the intentions of the brain, surgeons to put these things in place. It took a team of hundreds of people, thousands of people. I get all the credit like I did it. No, they did it. I was just a cog in the wheel.” (Nicole).

Rudi spoke about his participation in the Cybathlon and reported being overwhelmed by the amount of public interest and coverage of this event. The arena where the competition took place was sold out, the event was broadcast on television, various camera teams and journalists addressed him during and after the competition and he could see himself in the event’s promotional videos. In correspondence to that, he also appreciated the public forum that displayed the abilities of people with physical impairments. When asked what the Cybathlon meant to him, he responded:

“A lot. A very very lot. […] For a start, for my ego, definitively. […] To demonstrate, hey, one wheelchair user is not like another. We also are able to achieve a lot. […] To simply create this awareness! Simply, that people with disabilities are also people who also want to have a life and to get some publicity and not to be marginalized.” (Rudi).

For Rudi, the Cybathlon had a twofold meaning. First, he got recognition and received publicity for what he was doing. Second, he was able to create awareness for people with disabilities. This point was also stressed by another user:

“Cybathlon, I think it’s a good meeting to bring some awareness on this type of issue.” (Robert).

Neil, for example, got a personal handshake from the US president, which for him was social recognition on its highest official level. This was due to his BCI performances with implanted electrodes which no one else had before him.

These events or incidences create publicity and show the public what people with disabilities are able to achieve and to present them in the media and the public.

Also beyond the training, BCIs offered opportunities for social engagement and participation. One user creates artwork using her home-based BCI and displayed her pieces at national and international art exhibitions. Another user was given the opportunity to become a published book author (the book is about her, including her experiences as a BCI user; it was not written using a BCI).

“After working on the brain-computer interface for a couple of months, I had my attendant bring up the book [she had been writing before] on screen. I started revising it and editing it. Eventually, I published it […] I became a published author. That was more feeling of empowerment and ‘Look what I can do!’” (Nicole).

Nicole also gives talks about her experiences with BCIs, which is also important to her.

“I’m left with a feeling of ‘I achieved a lot’, but more importantly, I was able to make a contribution. I used to do charity work and I had to stop that when I lost my physical abilities. To be able to do something to make a contribution again was very important to me, very meaningful.” (Nicole).

Having had the “job” of BCI training as a meaningful occupation, which at the same time was conceived of as making a contribution, it was important for Nicole to find another occupation that was “meaningful”.

Other users generated personal homepages and provided online videos to inform, raise awareness and display their accomplishments.

Having a meaningful occupation and being part of a (work) team, getting social recognition, and taking part in public life, if not even creating publicity oneself, form part of having a social life. They are dimensions of social participation. As we have seen above, some interviewees appreciate these social dimensions even more so than the immediate effects of BCI training, such as feeding oneself with a robotic arm or being able to play a computer game. This was mentioned in particular from interviewees who led a life without disabilities before they had an accident or an outbreak of a degenerative muscle disease. The BCI training offered them some kind of *normality*, i.e. situations and circumstances they were used to before their impairment. This may be due to having a job again; being able to paint again, which was a hobby before the outbreak of the disease; playing games as you were used to; or getting out of the house again as before.

The importance of having opportunities for participation is underlined by the accounts of users we talked to who were born with some degenerative muscle disease. They all still have some residual muscle control in virtue of which they steer joysticks, control eye trackers or make use of voice decoders. As these technologies are more efficient than BCIs, these users are happy to stick with their current technologies. By means of these technologies they can engage in sports such as E-Hockey or Paralympic boccia, which play an important role in their lives. Hence, this may suggest that it is important for BCIs to also provide opportunities for social activities that can serve as meaningful occupations for their users.

#### Self-definition

“My brain had not forgotten.” (Nicole).

During the interviews, our study participants made various self-descriptions and -positionings. The reference point for these was either other people (without disabilities) or their former self, their future self or themselves vis-à-vis the machine/technology or their bodies. All these aspects have an impact on the present self and the way you see yourself.

Users that had an accident or an outbreak of some degenerative disease like amyotrophic lateral sclerosis (ALS) compare their present situation to their life before, their *former self*, so to say. In terms of independence, they point out activities that they cannot do anymore and activities that they regained with the help of the BCI. These include activities like painting or playing games, which are perceived as raising their quality of life.

“But we started doing just, like, computer control (…) like moving a cursor on a computer to do (…) some stuff (…) like Microsoft Paint. I’ve done some drawings and I’ve played a couple of video games and that part which was a big part of, you know, what I’m interested in, is playing video games, that could give me a lot of freedom to do what I want back.” (Neil).

Thus, the BCI allowed some to take up former habits or hobbies and to create a common thread in their biographies. As noted, Nicole was very appreciative of having the “job” of BCI training, which suits her personality in that she regards herself as a very busy and industrious person. Rudi sees himself as a very ambitious and competitive person and therefore highly appreciated the opportunity of competing in BCI gaming contests. Neil also regards ambition to be part of his personality. Hence, he also highly valued the possibility to live out this trait of character in BCI training.

“[…] one of my favorite tasks to do, because I have, you know, a set time to be, like, I have the goal and I want to get the goal, I want to be, you know, the best in it […] But it’s another thing where I have an actual score and I want to beat the score and I want to be the best and I want to be, you know, it’s one of those things, like, I want, I want whoever comes after me to go, like, oh he did it 25 times in two minutes, like, I need to really push myself to do more than that. That’s a big deal.” (Neil).

Users who were born with a muscular disease drew *comparisons to other people* rather than to their former selves.

Taking the example of Walter, he was concerned with “very abstract things like ‘What occupation shall I have?’ or ‘How shall I live?’ up to daily and small things like ‘What shall I eat today?’ and ‘At what time shall I go to bed?’ or ‘What shall I listen to on the radio?’” (Walter). According to him “[t]hese are all questions which may not be present to non-disabled people, because they are automatically self-determined in most cases.” (Walter).

Non-disabled people may not be aware of experiences and restrictions that people with disabilities are confronted with. Walter, in contrast, is very aware of the things he is able to do on his own and things he needs assistance with, which results in a different perspective on his life that is in contrast to non-disabled persons.

“Perhaps, I’m more aware of my self-determination or I perceive it in a more conscious way what a precious thing it is.” (Walter).

As these users need assistance for doing particular things, caregivers and relatives play a crucial role in their lives. As they also “have a say” in decision-making (Walter), which can consist of “making suggestions” (Mrs. Edlinger), it is important in particular to “find compromises both sides can live with” (Karl).

In terms of the BCI training, caregivers and relatives are affected primarily by getting the users to the locations of the BCI trainings. In one case the user had a home-based BCI, which required some basic knowledge about the BCI in terms of set-up and handling on the side of the caregivers.

Another reference point for BCI users is their *body*. BCI training can elicit various body-related experiences. These may be completely new body sensations or sensations users are familiar with but lost due to their accident or disease. Robert, who lost his ability to walk, describes the effect of the BCI training:

“It was something very important for me because I know I could see my legs before, but it was something that didn’t exist in myself. It was a very strange feeling before the experience and therefore I am more reconnected maybe not with a computer but with myself. Although it doesn’t move, it doesn’t move, but it belongs to me [laughs] but it’s strange what I say.” (Robert).

This experience helped Robert to “reconnect” to his body and to reincorporate his legs in his body image. Rudi also reported new kinds of body awareness: “I got to know my body in a new way” (Rudi) and positive effects from the BCI training: “Better concentration skills, a better sense for my body in some way, too.”(Rudi).

Nicole describes a strategic change in her thinking while using a BCI:

“I realized I didn’t have to try as hard as I was trying, that my brain had not forgotten.” (Nicole).

Nicole found out that her brain remained the same and did not lose any of its capabilities, as is shown when it is connected to exterior limbs (robotic arm). Regaining lost abilities or sensations can lead to a change in the users’ self-definition.

“Oh, God, it really changed my self-image. It changed, as I said, the empowerment. The feeling, ‘I did this, look what I can do.’ It helped me realize that -- I have a saying up on my wall, ‘You are more than the body you live in.’ I just realized the truth of that statement, that my brain was the most important part of me, and that working meant I could do a lot.” (Nicole).

Nicole redefined her self-image by prioritizing her brain. In that her brain has not changed and did not “forget” any of its capabilities, it is regarded as the most important part of her. It empowers her over her body, which is undergoing a degenerative process. By centering her brain within her self-image – we would like to call this *brainification* – Nicole describes something that can be seen as a typical worldview of the neurosciences. Melike Şahinol, having conducted extensive ethnographic field work on BCIs, portrays the practice of neuroscience by taking the example of BCI studies that give rise to a cerebro-centered view on the subject [34]. By detaching brain processes from body experiences and isolating cerebral entities from bodily movements in order to delegate cerebral motion activities to the computer, neuroscientists focus on the discrete data of brain activities. Body movements are seen as distorting these data. By transporting this dualistic view of brain data and body sensations, whereby the former are appreciated and the latter neglected, the user may also be affected. She may change the way she thinks of herself and her body.

Nicole reported a twofold change in her self-image, one creating a feeling of empowerment, the other giving rise to a centering of her brain (brainification). For Neil, BCI use “usually just feels like I am, you know, using a tool” (Neil). Other users displayed a more engaging relation to their BCI. Mrs. Edlinger sees herself in a “collegial” work relationship with the BCI, which “is actually more of a friendly helper than just a technical thing” (Mrs. Edlinger). While she is very appreciative of the BCI as her colleague and helper, it nevertheless does not change her self-understanding.

“I remained the same person also without the technology and Brain Painting enriches my life very much but has not changed who I am.” (Mrs. Edlinger).

As such, the BCI may be helpful in finding affirmation in who you are as a person and remaining unaffected by the BCI, no matter the benefit and joy it may provide.

Robert did some training in which he was supported by a virtual avatar that he learned to identify with. He refers to that when asked whether he somehow felt connected to the computer or the machine.

“Not at the beginning. After six or seven months yes, especially with the representation of the man on the screen. On this representation, I tried, I understood that the machine was completely outside of me, but I could control somehow something on the screen so it was for me quite interesting to be connected in this environment, I should say.” (Robert).

This “representation” or virtual avatar is sometimes used in BCI training to perform motions in a more effective and intuitive way. It is supposed to create some kind of imitating effect. Besides Robert also Neil and Nicole worked with it and they reported that particular exercises were easier when using this virtual input in contrast to only focusing on one’s own body. The feeling of becoming one with the avatar is called “presence” and has been tested in BCI studies that made use of virtual realities [35–37].

There have been reports of identification with the virtual avatar and of incorporating robotic arms into one’s body-image. Like elsewhere [38, 39], however, no remarks were made pointing into the direction of perceiving oneself as a hybrid or cyborg.

Nicole named the robotic arm that she was steering via BCI and started to incorporate it into her body- and self-image.

“Yes. I think it was the second day of training when [the robotic arm] became my arm. They stopped saying I move [the robotic arm]. Then I started saying, ‘I moved my arm.’ Not ‘Look what the arm did’, but ‘look at what I did.’ Instead of saying, ‘I moved the computer right’ I would say, ‘I moved right’ because it became my arm. I felt like it was part of me.” (Nicole).

Taking these accounts into consideration it becomes apparent that there is a range of different relations and stances that users are taking on BCIs. They range from rather detached and neutral to appreciative and embracing positions. This variety of attitudes may be due to the different backgrounds and characters of the users, the effort the training takes, the level of difficulty or ease-of-use the users see themselves confronted with, as well as the hopes and expectations they have towards the technology and the training.

The future self also features in the users’ accounts as a reference point, may it be as a developmental process or concrete hopes that are verbalized.

Mrs. Edlinger describes her Brain Painting as follows:

“How I compose the paintings has become more complex and more complicated. I used to paint way faster. Now, it takes up to several weeks or months.” (Mrs. Edlinger).

The BCI creates room for creative and artistic development. As Mrs. Edlinger has one of just a few home-based BCIs, she can also look forward to having this progress and development continue.

Neil hopes to get the opportunity to visit Japan in virtue of his BCI achievements.

“I really want to go to Japan, like, like that’s always been one of my dreams, like, my whole life was to go to Japan. I was like, there’s got to be like a university or something they want to have, like a demonstration or something […] so I can go to Japan because that’s not, I mean, that’s not very feasible with the money I have since I don’t have a real job or anything and it’s expensive to get to Japan.” (Neil).

Of course, users also hope for positive effects of BCI use in the future. Robert talks about the potential benefit of having a BCI at home.

“[…] it could be great for me to at least on your own environment, in your own home to be able to have more autonomy and BCI can contribute to that.” (Robert).

BCIs can be seen as transporting various hopes and potentials, allowing for development, whether it is in terms of physical improvements or in tasks such as painting or getting high scores in BCI games or other applications. It can be seen as something that can potentially improve your life situation or quality of life or may be a realistic vehicle for achieving some life dreams such as getting to visit Japan. All users expressed hopes and expectations that the technology will continue to improve and develop further. This includes users who do not depend on BCIs, because they are still able to use different technologies that work more efficiently, but may want to use BCIs in the future, especially in the case that their condition will deteriorate. It also encompasses users who already benefit from BCIs in terms of a progressing rehabilitation or in being able to perform certain activities and who hope for continued successful BCI use.

These various reference points determine processes of self-description and self-understanding. As such they impact the users’ *present self*.

As we have seen, BCI use can bring about “empowerment” (Nicole) and attributes meaning to the users’ doings. As mentioned in a quote above, Neil reported gaining “a lot of freedom” by being able to do the things you want to do.

Due to the relative recentness of the technology, which is predominately in its experimental stage, BCI users feel like “pioneers”.

“You were some kind of pioneer in this field and that was cool. No doubt, that was really cool. That was adrenaline, that was ego booster and so on.” (Rudi).

Neil also reported positive effects in terms of his self-esteem.

“[T]he whole thing is very humbling, but sometimes I’m like, yes you want to know, like, how cool I am, like, I’ll tell you how I’m doing all this cool stuff. And I met President Obama and that was, that was pretty awesome. So for that week I’m kind of, felt a little inflated in my self-worth.” (Neil).

In conclusion, BCI use elicits processes of self-definition among BCI users, no matter how diverse these may be in terms of their significance, depth and reference points. This diversity can be expected regarding the heterogeneity of the participants in terms of their backgrounds, physical conditions, expectations, opportunities, consequences as well as the aim and content of the respective BCI applications they were using. These self-definitions can result in changes, such as your self-understanding now in contrast to your past and former self or a changed body- and self-image, or in affirmations, such as self-assessments vis-à-vis the BCI or other (non-disabled) people.

## Discussion

Reflecting on the categories outlined so far, it can be stated that BCIs can maintain or recover a sense of agency, can provide opportunities for participation and can have positive effects on the users’ self-image. As such, BCIs are powerful tools for users who can benefit from them, helping them to retain valuable abilities or to improve their living situation. Hereby, the three categories are intertwined in various ways.

A sense of agency, i.e. the feeling of being an agent, plays a pivotal role in our self-understanding and BCI users also report that feeling in their self-descriptive accounts. You cannot claim ownership or be proud of actions that you do not feel responsible for. As mentioned above, BCI use can elicit empowerment and foster self-esteem. The users also stressed the importance of contributing to medical research and progress in curing or treating various diseases for future generations. BCI research also has a participatory component in that users stressed that they are part of a team.

The BCI also offered its users opportunities to participate beyond the research, which had an impact on their self-image and played a part in their self-definition. The immediate successes and achievements of the BCI training, such as feeding yourself or creating artwork, can also translate into further participatory opportunities (e.g. realizing exhibitions that display BCI-generated artwork, taking part in competitive BCI races or writing books about your BCI experiences). (The opportunities for participation provided by BCI use of course are not limited or inherent to the BCI technology.) These, at the same time, are things that are attributed to the users as individual achievements. Accordingly, this also strengthens the users’ sense of agency. Feeling like the author of one’s actions, having a social life and taking advantage of opportunities of participation, and making experiences that challenge one’s self-definition, maybe even ones that foster self-esteem, freedom and empowerment, can be regarded as very typical aspects that characterize human lives. Touching on these aspects, BCI use may be more than just testing a technology, but instead also affect users as a whole. BCI use poses questions in terms of one’s self-perceptions, the perception through others and resulting reflections on one’s range of human experience. These aspects may be relevant in particular for BCI users with physical impairments. This is reflected in BCI usage which allows for agency, participation and self-definition processes, but at the same time brings about its own challenges. BCI actions leave room for uncertainties on the side of the user. Participation opportunities can be confined to the period of the BCI study and could decline if BCI research loses its pioneer status. BCIs can reconnect users with their former life experiences, give them self-esteem or empowerment, but may also be an additional burden for caretakers or produce high expectations [23, 40, 41].

The challenge of dealing with emotions while operating a BCI has not been assessed yet in the literature. Suppressing one’s emotions, it can be argued, deprives us of a vital part of our human experience. At the same time it makes it difficult to fully embrace the technology as a user. BCI users regard the opportunities for participation and communication as social needs and are willing to take on certain risks (surgery, frustration, unfulfilled expectations).

BCI users do not question their human nature, i.e. fearing that technology will lessen their human integrity at the expense of turning into some wo*man-machine hybrid. As noted, Mrs. Edlinger explained that the BCI use did not change who she was, because she “remained the same person.” In the original text, in German, she actually used the word “human” and not “person”. What characterizes the users’ lives is a certain level of dependence on assistance, either in the form of technology or other people, caregivers and relatives. As such more independence and autonomy are things that BCI users seek. Technology is accordingly regarded as a potential to get there and is seen overly positive. To engage in more technology use, or even a “cyborgization” [16] is therefore seen as an opportunity and not a threat. Neil, for example, explained:

“He [Elon Musk] wants, you know, everyone to become cyborgs. I was like, well I’m doing my part, […] So if in the future I was presented an opportunity to do something like this again, I would.” (Neil).

Each category discussed above portrays a field of openness, possibilities, contingency and uncertainty where BCI users can make choices and decisions. They generally opt to see themselves as agents even though the situations – as we have seen in the case of failed actions – are often complicated and unclear. BCI training can be regarded as a deserving task and meaningful occupation in itself. At the same time it can be made use of in terms of taking advantage of these BCI achievements in various ways (exhibiting BCI-generated art, giving talks or writing books about the BCI experience). The realm of self-definition is wide and reference points are plentiful. For some it became a place to gain empowerment, freedom, self-esteem and confidence.

As a consequence, where BCI use offers opportunities in promoting or enabling particular desirable or valuable qualities, they are (often) seized by its users and the benefits BCI can bring about are clearly regarded as outweighing its risks.

## Limitations

The study contains several limitations. While the small sample number is not particularly at odds with the standards of qualitative research methods, we did aim for a higher number of participants. As it was particularly difficult to get into contact with BCI users, i.e. participants of experimental BCI studies, we could not manage to recruit more participants.

The sample of the study consists of persons that vary in many respects. Their physical impairments and diagnoses as well as the BCI type or model that they were using vary greatly. The heterogeneity of the sample illustrates a broad spectrum of relevant aspects that are related to BCIs. More detailed research with particular groups is required: users with the same physical impairments and users that work with the same BCI model.

This study limits itself to the perspective of BCI users with physical impairments. Other BCI stakeholders such as researchers, designers/developers, healthcare professionals, relatives and caregivers of users as well as non-impaired BCI users hold their own perspective, which needs to be researched in its own right. More detailed empirical research in these regards is encouraged.

## Conclusion

BCI users with physical impairments regard the potential of BCIs very highly. While they may not be applicable to all at the moment, they see potential for the future and stress the importance of investing in the development of this technology. Being an agent is an essential aspect of BCI usage and includes some level of uncertainty regarding the agency in place. BCI use also poses opportunities and challenges regarding the users’ self-definition. The users appreciated the possibilities for social participation that BCIs made available to them. These aspects stand for negotiation processes that take place in the face of perceptions about what are deemed to be valuable and desirable qualities. In awareness of the benefits of BCIs, users accept risks such as surgeries, unfulfilled hopes or data theft.

We may conclude by stating: if technology is beneficial to users, for example in terms of agency, participation, and defining and working on ourselves, technology is welcome. Technology does not make us less human even though we increasingly incorporate it. Rather the opposite seems to be the case: this technology can retain or enable valuable qualities such as agency or social participation. Where technology is at odds with users’ common human experiences, as for example having emotions, the technology at best is engineered in a way that encompasses necessary modifications. The message that these analyzed interviews can pass along to the development of the technology is to keep and strengthen those aspects of BCI use that promote abilities that are regarded as valuable and at the same time to look for ways to improve aspects that are in conflict with common human experiences.

## Supplementary information

**Additional file 1.** Topic guide.

## Data Availability

The data sets generated and analyzed during the current study are not publicly available, because a significant proportion of them identify people and organizations. Anonymous subsets are available from the corresponding author on reasonable request.

## Abbreviation

BCI: Brain-computer interface

## Footnotes

1 For a discussion on the peculiarity of BCI actions, see Steinert et al. 2018 [42]. As mentioned before, we focused on (re-)active BCIs. We therefore rule out passive BCIs. There is an ongoing debate on whether passive BCIs are supposed to be defined as BCIs [1].

2 One user stated: “The conversion, sometimes it’s good, sometimes it’s bad” (Rudi). The original word used in German (“Umsetzung”) refers to implementation or conversion. We opted for the latter, because in this context BCI thoughts are translated or converted into measurable signals that generate a specific output.

3 This relates to what Werner Rammert calls an “unmediated instrumental relation”: “As long as technologies, such as simple tools and machines, can be characterised as passive or semi-active means, they are used in an instrumental mode: People take and handle them to attain their goals at work or in other everyday life situations. The effective action of a tool or a machine is incorporated in its design, like the hammer’s long shaft and heavy weight at the end or the engine’s encapsulated explosion and the spark generated by the turn of the key. Therefore, the user can integrate these objects as mere instruments into his action” [43]. According to Rammert, this view is not adequate for advanced technologies like BCIs anymore. Nevertheless, we regard this view as still being dominant in our everyday understanding of technology.

4 It has been demonstrated that individuals tend to overestimate their level of control of actions [44–46]. Gino et al. [47] show that this also holds true for the opposite case, i.e. underestimating one’s level of control when there was a high level of control. The authors conclude that individuals tend to misjudge their control of actions in general. Van de Laar et al. [48] test the sense of control in BCI games which, however, were operated manually and not via BCI. They could neither observe an overestimation nor an underestimation of control among the test persons. Research has shown that individuals tend to overestimate control in particular when the action output is in line with their intentions, as “once an intention-to-act has been formed, actions and action consequences are more likely attributed to oneself even if they were externally generated” [49].

5 The Cybathlon is a tournament in which people with physical disabilities compete in various disciplines using state-of-the-art technical devices. It was organized by the Swiss Federal Institute of Technology in Zurich and took place in Zurich in 2016. The Cybathlon comprises six disciplines: races with arm and leg prostheses, exoskeletons, wheelchairs, Functional Electrical Stimulation bikes, and BCIs. Teams that compete with each other each consist of scientific-technical staff and one person with physical impairments that takes on the role of the technology’s pilot. The next Cybathlon is scheduled for 2020.
