# Supplementary material for: What is it like to use a BCI? – insights from an interview study with brain-computer interface users
Source: BMC Med Ethics. 2020 Jan 6;21:2. doi: 10.1186/s12910-019-0442-2 (PMC6945485; doi:10.1186/s12910-019-0442-2)
Supplement: Supplementary file 1 — Additional file 1. Topic guide. [file 12910_2019_442_MOESM1_ESM.docx]

Additional file 1: topic guide

This is the topic guide used for the interviews. It should be stated that each interview turned out to be different and that the topic guide was adjusted accordingly, either before or during the interview. As some users provided extensive accounts on the opening question, this made room for several respective follow-up questions. About some users there has been information available online which was included in the topic guide leading to some additional questions for the respective interviewee. The three users with speech impairments were interviewed using a shortened topic guide.

Topic guide

Personal background to understand the context:

- Personal situation of your condition? How did it come about that you participated in a BCI study?
- In your daily routine: what things do you need assistance with and which things can you do on your own?
- What role does technology play in your life? Before and now?
- What is your understanding of autonomy/self-determination and can BCI contribute to it?

BCI-related aspects:

- How and when did you hear about BCIs for the first time?
- What were your initial expectations of BCIs?
- For how long have you been operating a BCI or how many BCI training sessions have you had?
- How does a typical BCI training look like?

Self-experiential aspects of BCI use:

- How does it feel like to operate a BCI?
- What do you think of when operating a BCI? (What is your mental strategy?)
- Is the BCI doing what you want it to do?
- Has the BCI done things you didn’t intend it to do? (What is the reason when it doesn’t work?)
- Do you feel responsible for the BCI generated output?
- Do you need to adapt yourself to the computer or is it the other way around? (Both/neither?)
- Do you feel somehow connected to the BCI?
- Do you feel enabled/empowered or rather estranged/alienated when using a BCI? (Both/neither?)
- Has the BCI experience somehow changed your self-image?

General:

- What are the pro and cons of BCIs in comparison to other technological devices?
- If you could wish for improvements: what would it be?
- What do you wish for in the future and can BCI contribute to that?
- Is there anything else, you think is relevant and we haven’t touched on so far?
